# Training the Trainees in Radiation Oncology with Telemedicine as a Tool in a Developing Country: A Two-Year Audit

**DOI:** 10.1155/2011/230670

**Published:** 2011-04-26

**Authors:** Sushma Agrawal, Anil Kumar Maurya, Kirti Shrivastava, Shaleen Kumar, M. C. Pant, Saroj Kant Mishra

**Affiliations:** ^1^Departments of Radiotherapy, Sanjay Gandhi Post Graduate Institute of Medical Sciences, Lucknow 226014, India; ^2^Departments of Radiotherapy, Chattrapati Sahuji Maharaj Medical University, Lucknow 226003, India; ^3^Endocrine Surgery, Sanjay Gandhi Post Graduate Institute of Medical Sciences, Lucknow 226014, India

## Abstract

*Purpose*. The estimated new cancer patient load in the Indian state of Uttar Pradesh is 0.1–0.12 million per year. Approximately two thirds of these require treatment by a radiation oncologist. Radiation oncologists: cancer patient ratio in this state is 1 : 2000 as compared to the recommended 1 : 250. This problem is compounded by the poor infrastructure of radiation oncology departments in the state which is suboptimal for teaching, training of resident doctors, and treatment in most barring a few departments. To bridge some gap in the sociodemographics stated above and enhancement of training of residents, we submitted a project for establishment of a telemedicine facility in our department to the Department of Science and Technology, Government of India. We present the design, implementation, and a two-year audit of our tele-education activities. *Materials and Methods*. After the sanction of the project, we established telemedicine linkage with another medical institute in the city located 25 kms away in 2007. After implementation of the project, academic sessions designed for trainee residents in our department were shared with the remote end. A record of these activities and a feedback of the activities were audited at the end of 2 years of implementation of this project. *Results*. Regular videoconferencing sessions comprising of lectures on clinical oncology, medical physics, and radiobiology were held. Feedback from the users revealed satisfaction with the content of the academic sessions for the purpose of MD training. *Conclusions*. Distance education in radiation oncology is an important tool for training of the trainee residents.

## 1. Introduction

Cancer is the second leading cause of death in most developed countries today [1]. Developing countries are gradually following similar trends in cancer incidence as the Western world. The projected number of new cases by the year 2015 is 5 million for the developed countries and 10 million for the developing countries, while the number of deaths estimated will be 3 and 6 million, respectively. In India, the state of Uttar Pradesh (U.P.) has a population of 150 million, and it was estimated that annual new cancer patient load, would be around 100,000 to 120,000 by the year 2010. To cater to this load approximately 200 radiation oncologists are required as compared to only 50 existing in the state at any given time [2]. International Atomic Energy Agency (IAEA) has suggested the desirable requirement for radiation oncologists as one per 200–250 cancer patients [3]. 12–15 medical graduates (MBBS) enroll for the postgraduate training program in radiotherapy (MD radiotherapy) in the state of U.P. per year, and not all the radiotherapy departments are fully equipped with the basic radiotherapy facilities and faculty for effective teaching and training. To cater to the increasing load of cancer patients, creation of adequate and skilled manpower to run oncology centers effectively is the need of the hour. Training in radiation oncology requires teaching and training in radiation physics, radiobiology, clinical oncology for which resources are lacking. The basic radiation treatment facilities exist in a limited number of medical colleges, and a few private cancer centers in the state, whereas advanced facilities exist only in our department, being a part of a tertiary level medical institute and a regional cancer centre. In order to fulfill the ever increasing demand of competent radiation oncologists, telemedicine can be utilized to disseminate knowledge and skills to less privileged centers to complement their existing system. 

Applications of telemedicine in radiation oncology have been reported by various authors. Olsen et al. has discussed the requirements and applications of telemedicine in radiotherapy treatment planning [4]. They have described three levels of telemedicine application in radiotherapy treatment planning. Level 1 features video conferencing for consultation and display of radiotherapy images and dose plans. Level 2 involves replication of selected data from the radiotherapy database—facilitating remote treatment planning and evaluation. Level 3 includes real-time, remote operations, for example, target volume delineation and treatment planning performed by the team at the satellite unit under supervision and guidance from more experienced colleagues at the main clinic. Menezes et al. has discussed the use of telemedicine in oncology settings [5]. They have shared their experience with National Telesynergy Network for Oncology Services in Ireland which was established with a aim to improve service delivery and efficiency, reduce patient travel, and support earlier diagnosis. Keishiro Suzuki has demostrated the development of a web-based, remote radiation treatment planning system which allowed staff at an affiliated hospital to obtain support from a fully staffed central institution [6]. In a study by Maguire et al., telemedicine was shown to bridge the academic and community radiation oncology treatment planning [7]. They have demonstrated that complex radiation treatment planning review was feasible between an academic and community hospital via telesynergy network with a high level of physician satisfaction. Though use of telemedicine for distance education is common, it has not been reported in the field of radiation oncology.

In the XIth National Cancer Control Program of Ministry of Health and Family Welfare, Government of India, the experts have recommended closure of MD radiotherapy programs at ill equipped centers [2]. With a view to facilitate teaching, training, and carry-out collaborative clinical and translational research of the common cancers seen in the state of U.P., we embarked upon a project to establish telemedicine link with other departments of radiotherapy of the teaching medical institutions in the U.P. having limited resources in a phased manner. Ours is a tertiary level teaching hospital with a fairly well equipped radiation oncology department in the context of a developing country. The objective of the first phase of this project was to establish links with the Chhatrpati Shahuji Maharaj Medical University (CSMMU) within the city located 25 km away from our institute and evaluate its benefit before embarking on expansion to other locations. 

## 2. Materials and Methods

### 2.1. Project Planning, System Study, and Design of the Teleradiotherapy Network

**Figure 1:**
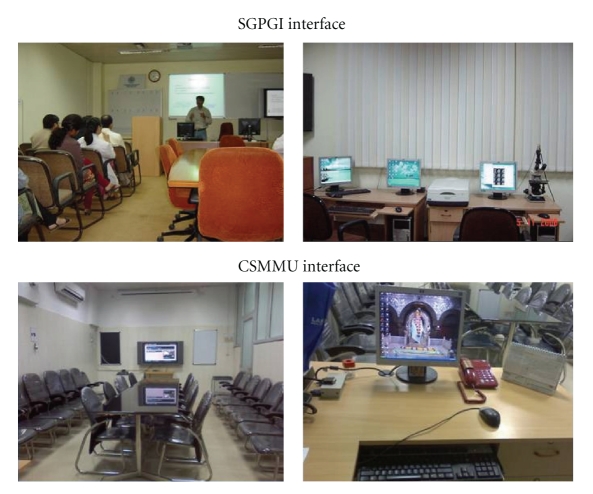
Picture of the telemedicine setup at the primary and remote node.

In 2003, a presentation was made by our department to the Department of Science and Technology (Govt. of India), regarding the healthcare needs pertaining to oncology in the state of Uttar Pradesh. The members were apprised of the usefulness of telemedicine as a tool to disseminate knowledge and skills to various teaching hospitals of the state to produce competent specialists, for teleconsultation, for telefollowup of cancer patients leading to possible benefits for the health delivery system for the citizens of the state. The technical, medical, and financial issues relating to telemedicine were also deliberated. The project was approved in 2004. A grant of 75 lakh Indian rupees (approximately 1.6 lakh USD) was granted for this project towards cost of hardware and software including intradepartmental network. It was recommended that for phase I of this project, successful telelinks between departments of radiotherapy of SGPGIMS and CSMMU should be first established before establishing tele-links with medical colleges in other cities of the state. The department of radiotherapy of the CSMMU was chosen as the remote center for telelink up in the first phase of this project. With the help of the School of Telemedicine of our institute the concept was translated to designing and developing an exclusive Telemedicine Network for specific application for Radiotherapy. The organizational aspects both technical and administrative were addressed before implementing the project. The technical part of the project was implemented through technical partner, Online Telemedicine Research Institute, Ahmedabad (OTRI) and the network architecture, hardware and software for the telemedicine platform were worked out. Polycom (Model: VSX 5000) PAL camera-based video conferencing equipment and 42′′ Plasma monitor with a screen resolution of 1024 × 768 for display was installed at both the nodes. The software used to establish the video-conferencing connectivity is People + Content IP supplied by Polycom. An integrated services digital network (ISDN) telecommunication media (128 Kbps) with static IP was chosen for the network. The telemedicine platform so created at both the nodes is shown in Figure 1. 

### 2.2. Implementation of the Project

The project implementation team inspected the physical infrastructure at the remote center to ensure suitable environment for installation of the telemedicine platform. Room dimension, power situation, and acoustics were the few factors important for deciding site suitability. Project manpower appointed were 2 telemedicine technicians, one for host node and other for remote node. Other technical manpower support was provided by the School of Telemedicine of our institute as per the requirements from time to time. Site preparation and installation of equipments at both nodes were completed along with integration of ISDN telecommunication media in the month of February 2007. 

### 2.3. Testing, Demonstration, and Training

After the technical testing of telecommunication with ISDN lines, functional modules of telemedicine were tested and demonstrated to department faculty and trainee residents at the host and remote node. One week on-site training was provided to the telemedicine technicians which continued later with demonstration sessions. All above mentioned training and demonstrations were given by engineers and application specialists from project technical partner OTRI. 

From April 2007 to May 2009, regular videoconferencing sessions were conducted. The academic sessions for post graduate students conducted in the host department were shared in real time interactive videoconference mode with the remote participants being trainee residents and interested faculty. A total number of 201 academic sessions were planned to be transmitted in two years. These sessions consisted of lectures on clinical oncology, medical physics, and radiobiology delivered by either trainee residents or hospital services residents, medical physicists or clinical faculty as a part of the academic roster of the department. Any questions at the end of the sessions were attended by the presenter and the faculty from the host end. This network was also used for teleconsultations, live telecast of the proceedings of the national conference of radiation oncologists, and shared lectures of national as well as international guest speakers visiting the host department. The progress and difficulties faced in this project was reviewed regularly by both ends. Due to narrow bandwidth of ISDN network image, quality of image transmitted was poor, and there was significant time lag between audio and display signals. Subsequently, after one and a half years, the network was shifted from 128 kbps ISDN to 2 Mbps leased optic fiber circuit with a view to expand the activities and correct the above problems. 

### 2.4. Method of Audit

All the events taking place between the two telemedicine nodes were recorded in the log book maintained on day-to-day basis. Later, the data was entered into the prescribed proforma devised for the evaluation of the activities. Periodic audit report at the end of each year was submitted to the funding agency. Final evaluation of the project was carried out by compiling the data of the activities of year 2008-2009. 

### 2.5. Feedback

A feedback from the users at the remote end after each session was collected in the last 6 months of the audit regarding the content of the program and the quality of the videoconferencing. This was obtained on a questionnaire and the feedback received was recorded on an excel sheet. There were 4 questions in the questionnaire pertaining to the (a) quality of content of the topic discussed, (b) utility of the topic chosen for the purpose of MD trainee, (c) efficacy of the instructor to communicate, and (d) the quality of videoconferencing. Every attendee filled the feedback form and the responses were rated on a scale of 0–10. The data was then analyzed for the percentage of participant's response obtained for score more than 7. A score of more than 7 was chosen for evaluation since it reflects a better than average score on a qualitative score of 1 to 10. For the purpose of reporting, a feedback was later also obtained from the trainees whether they preferred in person lectures or videoconferencing. 

## 3. Results

The results are based on an analysis of the events recorded in the log book and feedback received from participants (Table 1). From April 2007 to May 2009, regular videoconferencing sessions were conducted. A total number of 201 sessions were planned to be transmitted in two years and amongst this a total of 119 sessions could be transmitted. The rest had to be cancelled for either technical reasons (20%) or nonavailability of the presenter at the host end (80%). 6 to 8 participants (trainee residents and in-charge faculty at the remote end and 16 to 20 participants (all trainee residents, hospital services residents, and faculty and medical physicists) at host end) attended the sessions regularly. The technical problems for cancellation of sessions were ISDN connectivity problems, videoconferencing equipment functionality, and disruption of power supply. Since the remote end itself is a medical university teleconsultation activities were very few and subsequently, the need for teleconsultation was not felt. After initiation of the project, feedback from the remote end revealed that the image quality was poor and there was a significant time lag between audio and video signals. Audio input was optimal to understand the intermittent discussions at the host end during the sessions, but had scope for improvement. This was attributed to narrow bandwidth (128 kbps ISDN network). Subsequently after one and a half years, the network was shifted from 128 kbps ISDN to 2 Mbps leased optic fiber circuit. The image quality of the videoconferencing improved significantly.

 Feedback of questionnaire revealed that the quality of content of the topics discussed was good (score assigned more than 7 by 76% users), topics chosen were relevant for the MD trainee (score assigned more than 7 by 77% users), presenters effectively communicated their presentation (score assigned more than 7 by 70% users), and the quality of videoconferencing was given a score of more than 7 by 60% users. The trainees were of the opinion that the topics covered were relevant and well discussed from the MD examination point of view. An average of 3 questions per session were received and attended by the presenter or faculty at the host end to satisfy the queries at the remote end. Most of the trainees at the remote end preferred in-person lectures to videoconferencing because of time lag and occasional disconnection of the transmission. 

## 4. Discussion

Telemedicine applications in radiation oncology are multiple which ranges from tele-education to assistance in simulation, radiation treatment planning, and dose calculation [4–9]. Distance education in radiation oncology is an important modality of knowledge sharing for creation of manpower trained to deliver appropriate treatment to cancer patients. Unlike many other fields of medicine, radiation oncology is a dynamically changing, high-technology field, and successful practitioners for the future need to evaluate and adapt to changing patterns of practice. Training in suboptimally equipped radiation oncology centers is inadequate for residents in training. Exposure of residents to available technology, its principles, usage, and outcomes is essential for enhancements of their theoretical knowledge. Though telemedicine for distance education is not a new concept its implementation in a radiation oncology setup in a developing country has not been reported earlier. In this paper the entire process of developing a telemedicine project such as concept, design, development, implementation, administration, and evaluation have been addressed in great detail to make the reader understand the practical issues while deploying such a project. Telemedicine fulfilled its expectations of delivering quality education to the trainee residents for their MD exams and fill the gap in of scarcity of teachers to train the residents. The method of assessment used by us to evaluate the benefit due to telemedicine still has scope for improvement. In due course we shall modify our questionnaire to elaborate the benefits with telemedicine. Also, we have now taken measures to ensure strict discipline on behalf of presenters at the host end to minimize human factors as cause of cancellation of the proposed academic sessions. This project has created awareness amongst teachers and trainees in other medical colleges in the state about the benefit of teleeducation and its success is evident from the fact that they are keen to establish telelinkage with our center. Further, we also intend to extend this facility for other applications of telemedicine in radiotherapy as discussed earlier. 

## 5. Conclusions

Distance education in radiation oncology is an important tool for training of the trainee residents. We were able to facilitate teaching and training of residents at the remote center as envisaged though cancellation of sessions should be minimized. Two years of experience has taught us lessons to be able to handle the technical challenges to further expand our network to other centers in the state. The project is now ready to be launched into the second phase. 

## Figures and Tables

**Table 1:** Subject-wise data of telemedicine sessions during the years 2007–2009.

Year	Clinical oncology	Radiation physics	Radiobiology
2007-2008	35	26	8
2008-2009	24	11	3
